# Supplementary figures and images for: Linkage Disequilibrium and Population Structure in Wild and Cultivated Populations of Rubber Tree (Hevea brasiliensis)
Source: Front Plant Sci. 2018 Jul 3;9:815. doi: 10.3389/fpls.2018.00815 (PMC6037771; doi:10.3389/fpls.2018.00815)

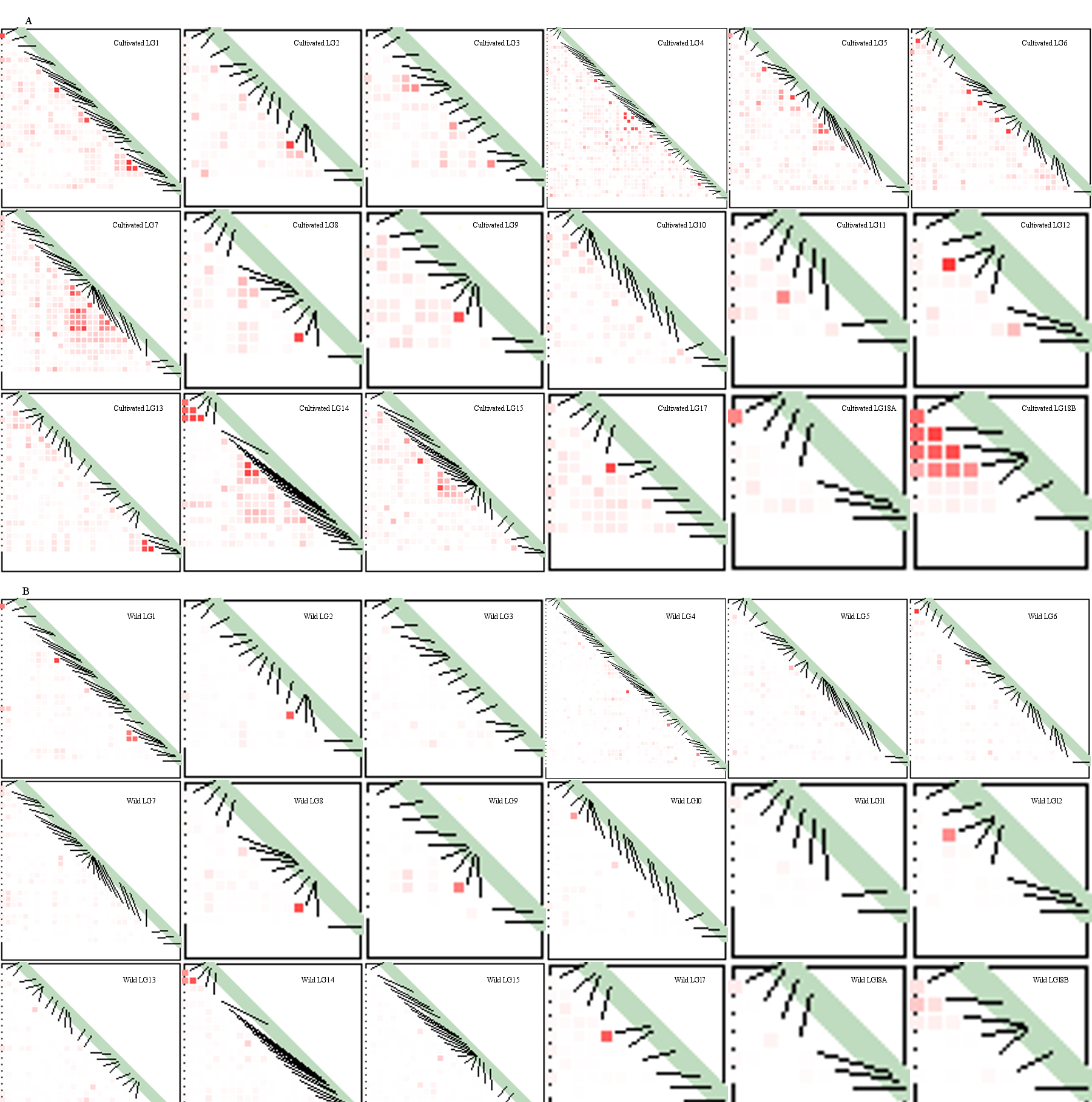

Supplement: FIGURE S1 — Plots of LD heat maps for (A) with 47 breeding accessions and (B) with 300 accessions from wild germplasms. The rubber tree LGs are represented by a diagonal bar. Markers were ordered on the x- and y-axes based on genomic location; therefore, each cell of the heat map represents a single marker pair. The r2 values for each marker pair are presented in the bottom half of the heat map and are represented by shades of red increasing in intensity in equal increments of 0.1 from 0.0 (white) to 1.0 (red). [file Image_1.JPEG]

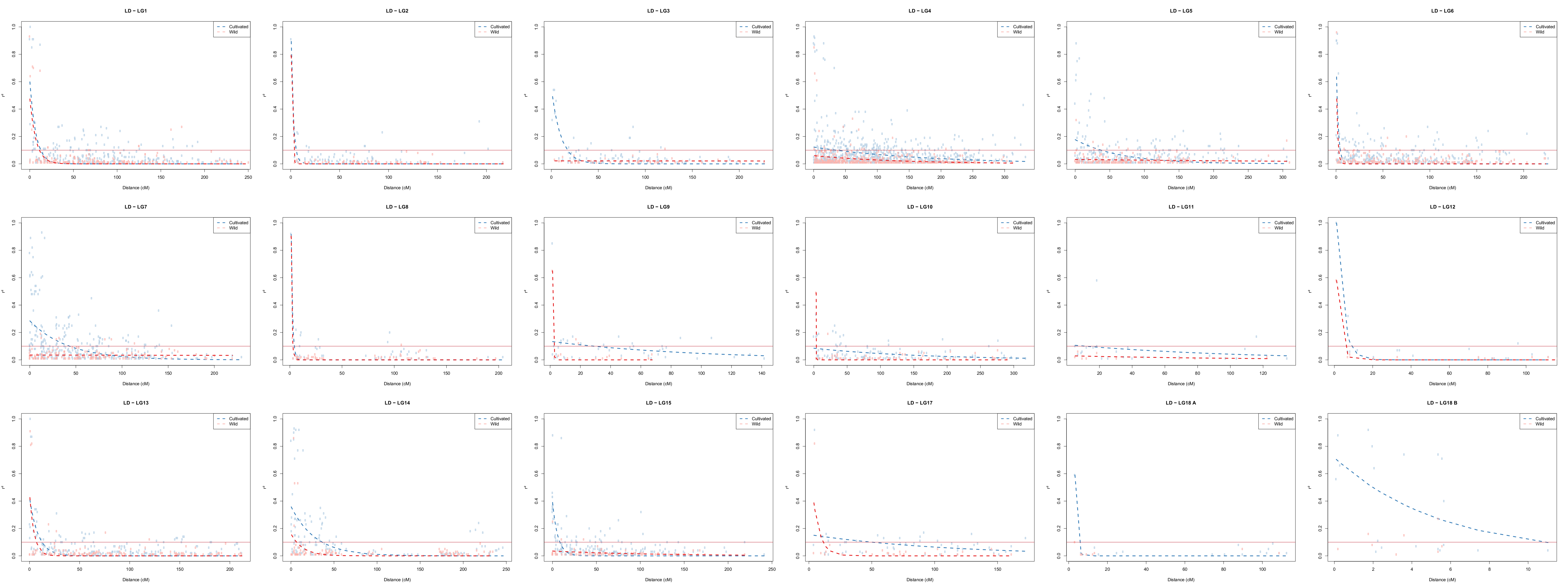

Supplement: FIGURE S2 — Decay of LD (r2) as a function of genetic distance (cM) between pairs of loci in individual LGs. Only r2 values with P < 0.05 are shown. [file Image_2.JPEG]
